# Double vertebral artery-connected persisting proatlantal artery coexisting with a persisting carotid duct in an adult case: first evidence

**DOI:** 10.1007/s00276-026-03892-0

**Published:** 2026-05-19

**Authors:** Mugurel Constantin Rusu, Adelina Maria Jianu, Laura Octavia Grigoriţă, Răzvan Costin Tudose

**Affiliations:** 1 Division of Anatomy, Department 1, Faculty of Dentistry, “Carol Davila” University of Medicine and Pharmacy, 8 Eroilor Sanitari Blvd., 050474 Bucharest, Romania; 2 Department of Anatomy and Embryology, Faculty of Medicine, “Victor Babeș” University of Medicine and Pharmacy, 300041 Timișoara, Romania; 3 Municipal Emergency Clinical Hospital of Timișoara, 300041 Timişoara, Romania

**Keywords:** Carotid duct, Ductus caroticus, External carotid artery occlusion, External carotid artery stenosis, Persistent proatlantal artery, Vertebral artery

## Abstract

**Purpose:**

The persistent proatlantal artery represents persistence of the embryonic first cervical intersegmental artery and creates a carotid–vertebral anastomosis. A persisting carotid duct is an exceptionally rare persistence of the dorsal aortic segment between the third and fourth aortic arches. It may be associated with segmental occlusion/stenosis of the proximal external carotid artery.

**Methods:**

Archived **c**omputed tomography angiography files in a 62 year-old man were studied anatomically.

**Results:**

A right-sided persisting carotid duct arising from the subclavian artery with short proximal ECA occlusion/stenosis and reconstitution of the distal ECA was found. A large type II PPA arose from the reconstituted ECA, formed a suboccipital loop giving rise to the occipital segment of the occipital artery, and established two distinct junctions with the vertebral artery: an anterior branch to the V3 segment near the posterior condylar foramen and an inferior branch to the V2 segment immediately before entry into the C1 transverse foramen.

**Conclusion:**

This configuration provides imaging evidence of a true ‘double-connection’ of a persisting proatlantal artery, coexisting with a persisting carotid duct and proximal external carotid occlusion/stenosis, with potential implications for carotid interventions and posterior-circulation haemodynamics.

## Introduction

Primitive carotid–vertebrobasilar anastomoses develop early in human embryogenesis and normally involute [1]. The proatlantal intersegmental artery accompanies the first cervical nerve and connects the embryonic carotid and vertebral circulations; persistence of this channel in adult life is termed a persistent proatlantal artery (PPA) [1, 2]. Two variants are typically recognised: type I, from the internal carotid artery (ICA) and type II, from the external carotid artery (ECA) [3–5]. In type II, the anomalous vessel courses posteriorly in the upper neck, often traverses the C1 transverse foramen, and then joins the vertebral artery (VA) before entering the skull [4, 5]. Although PPAs are frequently incidental, their recognition can be clinically critical because the posterior circulation may depend on the anomalous channel [4, 6]. The occipital artery may arise from a persistent proatlantal channel rather than directly from the ECA [1]. A comprehensive harmonised review of PPA types, subtypes, and classification frameworks, including the course-based system and Cohen’s spinal/occipital subtypes, has recently been published [7].

The carotid duct (CD, ductus caroticus) normally regresses [8]. Adult persistence is exceptional and may coexist with segmental occlusion/stenosis of the proximal ECA, with the persisting CD acting as an alternative feeder of the distal ECA [8]. Here, we report an adult case in which a persisting CD and proximal ECA occlusion/stenosis coexist with a type II PPA demonstrating two separate junctions to the VA (V2 and V3).

## Material and method

Computed tomography angiography of the head and neck was performed in a 62 year-old man using a SOMATOM Definition Edge CT scanner (Siemens Healthineers). Archived DICOM datasets were imported into Horos (Horos Project) for post-processing, including multiplanar reconstruction, maximum intensity projection, and 3D volume rendering. Vessel courses, external diameters and inter-vascular distances were measured on calibrated reconstructions. No patient-identifying information was reported. Therefore, the clinical details were not available. Terminology (‘persisting carotid duct’, ‘occlusion/stenosis’, ‘type II PPA’) followed prior anatomic and radiologic descriptions [1, 4, 8].

## Results

**Fig. 1:**
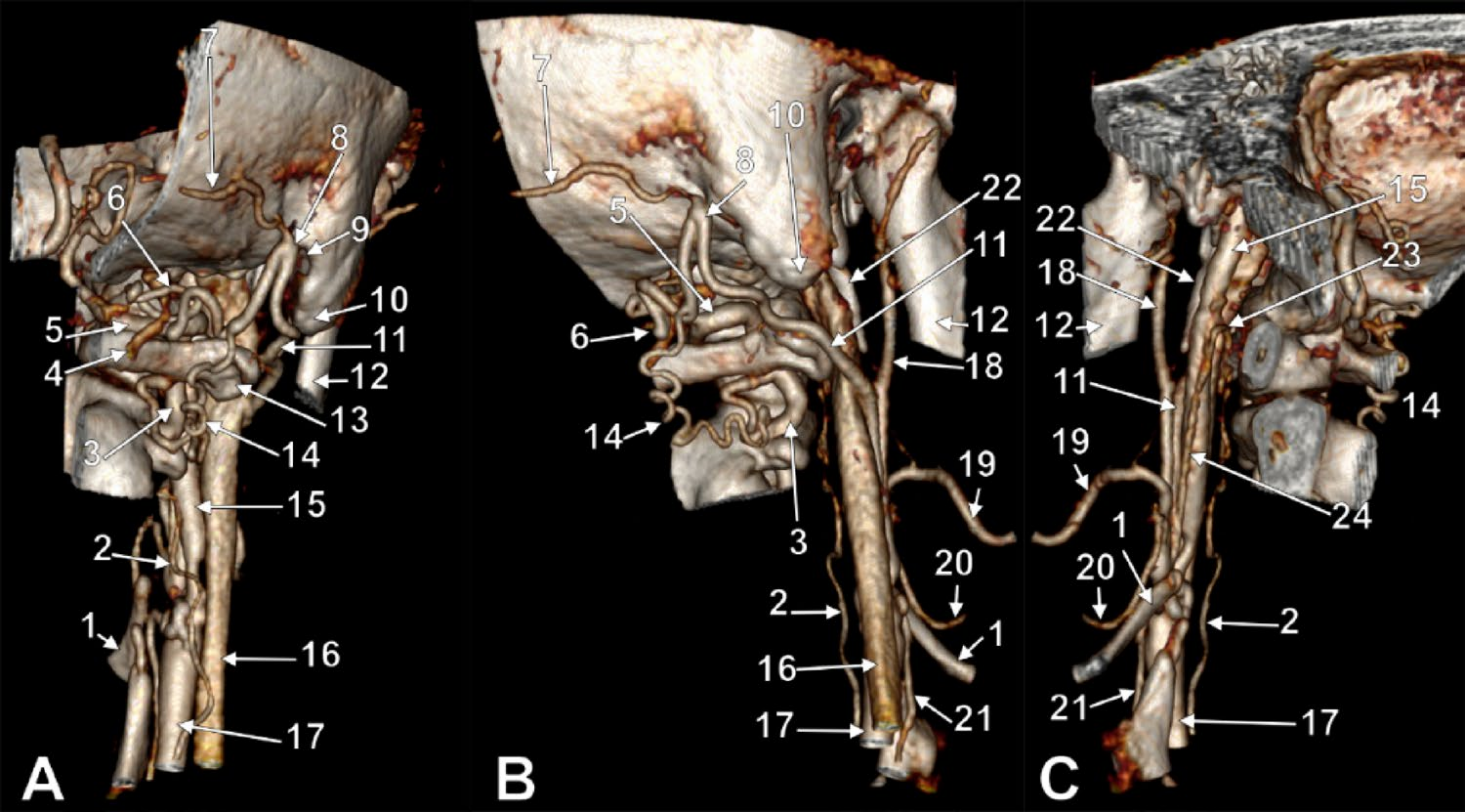
CTA 3D volume-rendered reconstruction of the right cervical and suboccipital regions showing the persisting carotid duct (PCD) and the persisting proatlantal artery (PPA). **A**: postero-infero-medial view; **B**: postero-infero-lateral view; **C**: medial view. (1) greater hyoid horn; (2) ascending PCD; (3) V2 vertebral artery (VA); (4) posterior condylar vein; (5) V3 VA; (6) anterior br.of PPA ; (7) occipital artery; (8) superior loop of PPA; (9) mastoid notch; (10) mastoid tip; 11. PPA; 12. mandibular ramus; 13. transverse process C1; 14. inferior br. of PPA; 15. internal carotid artery; 16. internal jugular vein; 17. common carotid artery; 18. external carotid artery; 19. facial artery; 20. lingual artery; 21. superior thyroid artery; 22. styloid process; 23. preatlantal loop of PCD; 24. descending PCD

**Fig. 2:**
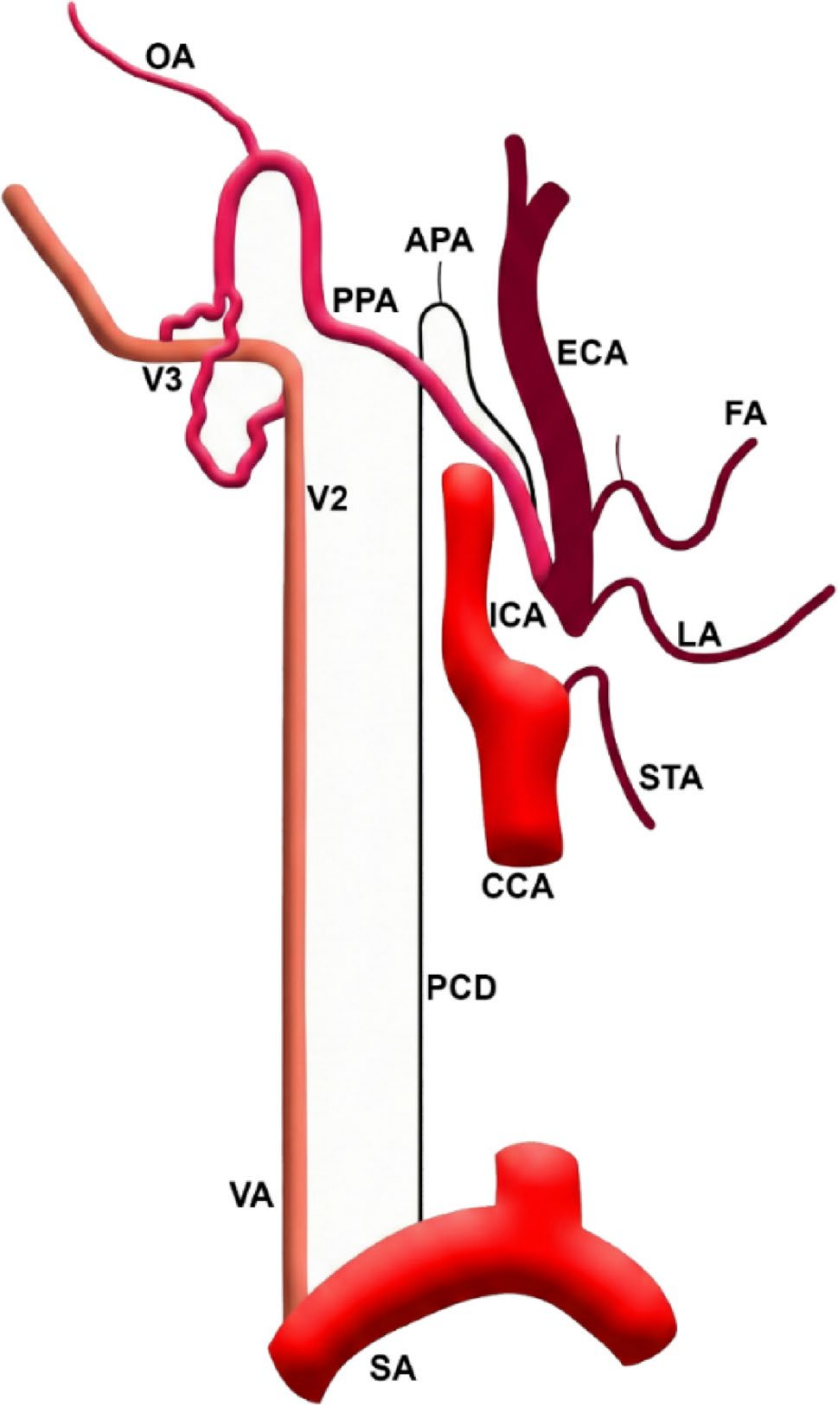
General diagram of the right-sided anatomical variants: persisting carotid duct (PCD) and proatlantal artery (PPA), a true ‘double connection’ of the same PPA to both V2 and V3 segments of the vertebral artery (VA) as two distinct junctions, and occlusion/stenosis of the initial segment of the external carotid artery (ECA). SA: subclavian artery; CCA: common carotid artery; ICA: internal carotid artery; STA: superior thyroid artery; LA: lingual artery; FA: facial artery; APA: ascending pharyngeal artery; OA: occipital artery

Multiple novel arterial variants were identified on the right side (Figs. 1 and 2).

**Fig. 3:**
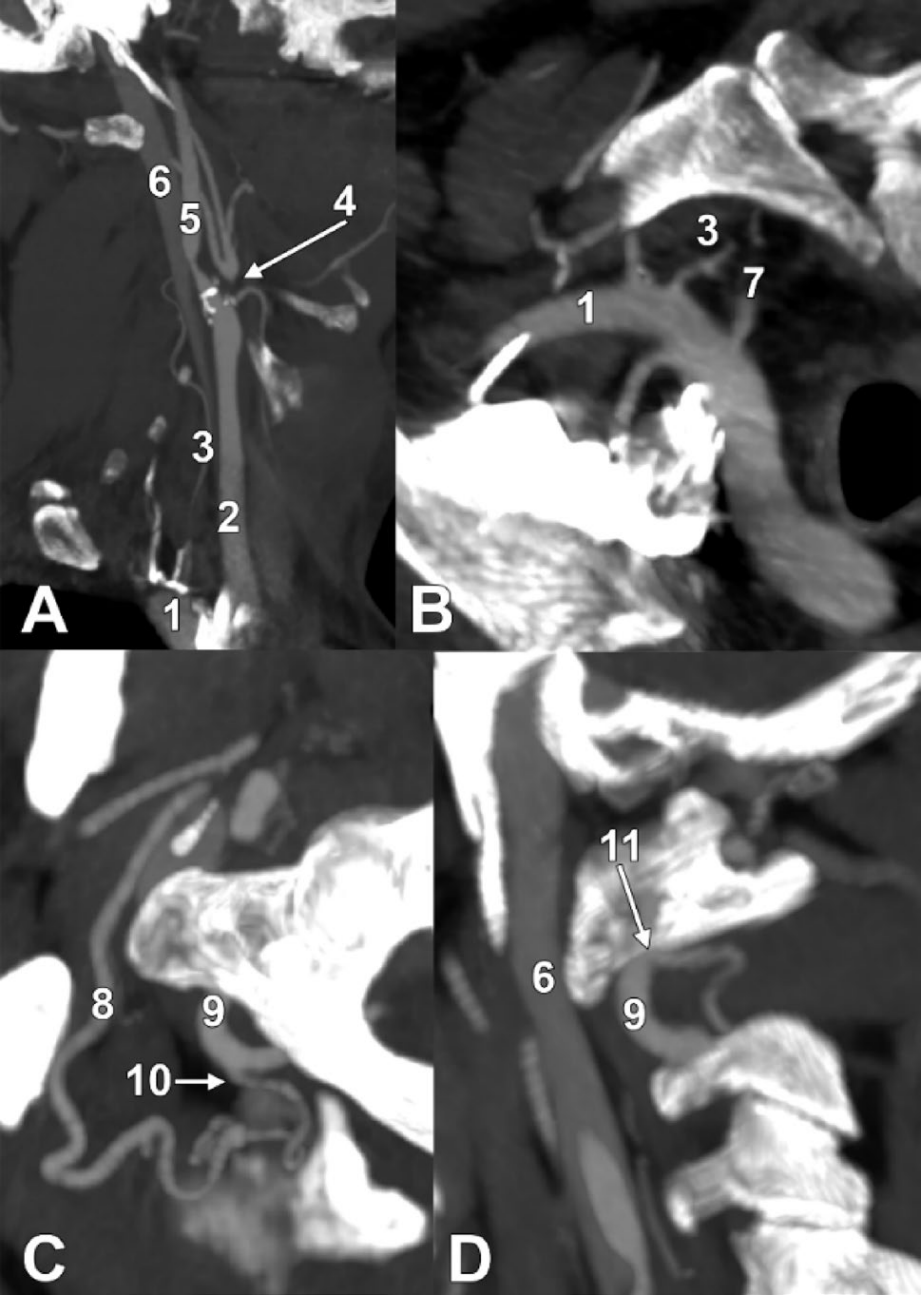
Oblique two-dimensional slices demonstrating details of arterial connections on the right side. **A** Oblique coronal slice through the right carotid axis, anterior view. **B** Oblique axial slice through the right subclavian artery, viewed antero-superiorly. **C** Oblique axial slice through the right atlanto-occipital space and the superior connection of the occipital and vertebral arteries, superior view. **D** Oblique coronal slice through the inferior connection of the occipital and vertebral arteries, anterior view. (1) subclavian artery; (2) common carotid artery; (3) carotid duct; 4. proximal external carotid artery non-opacification; 5. internal carotid artery; 6. internal jugular vein; 7. vertebral artery; 8. persisting proatlantal artery (PPA); 9. vertebral artery (VA); 10. superior PPA-to-VA anastomosis; 11. inferior PPA-to-VA anastomosis

On the right, the VA arose from the superior aspect of the subclavian artery, 2.01 cm distal to its origin. An 8-mm vessel arose from the subclavian artery 1.02 cm distal to the VA origin and ascended posterior to the internal jugular vein and CCA. It continued posterior to the ICA, reaching the anterior aspect of the root of the atlas transverse process, looped superiorly, then descended medial to the ICA to terminate at the origin segment of a large posterior ECA branch. This vessel was identified as a persisting CD (Fig. 3A, B). The ascending pharyngeal artery continued superiorly from the upper preatlantal loop of the persisting CD.

The CCA ended inferolateral to the tip of the greater horn of the hyoid bone. Its dilated terminal segment gave rise to the superior thyroid artery and the ICA. The ECA origin was non-opacified for 0.42 cm from the CCA termination, after which the ECA resumed as a distal blunt end, consistent with proximal ECA occlusion/stenosis (Fig. 3C).

From this blunt ECA end arose an 18 mm PPA that ascended obliquely posterosuperiorly deep to the digastric muscle, and crossed the atlas transverse process 34 mm inferior to the mastoid tip. It then looped superiorly to contact the mastoid within the aspect of the digastric/mastoid notch, 0.52 cm lateral to the occipitomastoid suture; the terminal (occipital) segment of the occipital artery arose from this superior loop.

The descending PPA limb reached 0.59 cm posterior to VA V3, both posterior to the atlas lateral mass, and was divided into anterior and inferior branches. The anterior branch joined V3 0.65 cm posteroinferior to the posterior condylar foramen, with the posterior condylar vein descending over this junction. The tortuous inferior branch descended posterior to the axis transverse process, then ascended to reach immediately inferior to the atlas transverse process and join VA V2 inferior to the C1 transverse foramen (Fig. 3D, E).

## Discussion

We demonstrated a right-sided constellation of rare cervical arterial variants: a persisting CD arising from the subclavian artery, associated with short proximal ECA critical occlusion/stenosis and distal ECA reconstitution, from which a large type II PPA arose, formed a suboccipital loop (giving rise to the terminal occipital segment of the occipital artery), and, most notably, created a true “double connection” to the VA via two distinct anastomoses, one to V3 (anterior branch) and one to V2 (inferior tortuous branch) just before the latter’s entry into the C1 transverse foramen, with implications for interpretation and procedural planning in carotid/ECA territory.

During early embryogenesis, six paired aortic arches transiently connect the ventral aorta to the paired dorsal aortae [9, 10]. The third aortic arches subsequently form the proximal ICAs, while the ventral root between arches III and IV elongates to become the common carotid artery [10, 11]. The CD refers to the dorsal aortic segment interposed between the third and fourth arches; under normal development it involutes as the CCA consolidates and the definitive carotid configuration is established [10, 11]. Persistence of this segment is implicated in rare carotid branching patterns.

Type II PPA is described as arising from the ECA and joining the VA before intracranial entry [4, 5]. The key novelty here is a single PPA forming two distinct junctions with the VA (V2 and V3), whereas standard descriptions usually depict a single PPA–VA connection [4, 12]. Embryologically, the VA forms via longitudinal anastomoses between adjacent cervical intersegmental arteries; persistence or incomplete fusion can yield duplication/fenestration and complex cervical anastomotic patterns [1]. Accordingly, the additional V2 junction may represent persistence of an accessory longitudinal connection between the proatlantal channel and the preforaminal VA, rather than a secondary postnatal collateral.

Although an exact “double VA–connected PPA with persistent CD” has not been previously reported, a developmental linkage is plausible: if a large proatlantal channel persists, it may arise from/near the ductus caroticus, and Lie (quoted) suggested that a prominent PPA could “stimulate” CD persistence [1]. The coexistence of persistent CD with proximal ECA occlusion/stenosis further supports this theme, resembling a prior adult pattern of subclavian-origin CD supplying a distal ECA above a short proximal ECA occlusion/stenosis [8]. Clinically, both variants are relevant: PPAs can render carotid/ECA procedures hazardous for posterior circulation [4, 6], and a persisting CD may be the critical feeder of ECA territory when proximal ECA is occluded/stenosed [8].

The differential diagnosis of the proximal ECA non-opacification merits explicit consideration. Volume-rendered CTA (Figs. 1 and 2) displays only intraluminal contrast and cannot depict mural thrombus, calcified plaque, or low-grade luminal irregularity. In a 62 year-old man, atherosclerotic critical stenosis/occlusion at the carotid bifurcation is mechanistically plausible and haemodynamically consistent with the observed collateral reconstitution pattern: the persisting CD, a developmental variant already present, would also function as an acquired collateral route rescuing the distal ECA territory. We acknowledge this limitation and accept that the 0.42 cm proximal ECA non-opacification most likely represents acquired critical stenosis/occlusion rather than true congenital segmental agenesis. Regardless, the anatomical configuration and clinical implications remain unchanged: the persisting CD is the critical feeder of the distal ECA, and the type II PPA arising from the reconstituted ECA constitutes the dominant collateral to the vertebrobasilar circulation. The morphological hallmarks are inconsistent with simple acquired collateralisation via VA muscular branches: (i) the vessel arises from the reconstituted distal ECA, not from the VA itself; (ii) it forms a well-defined suboccipital loop from which the terminal occipital artery arises, documented for both type I and type II PPA including the occipital subtype [7]; (iii) it anastomoses with the VA at two separate points (V3 and V2); and (iv) the coexisting persisting CD provides additional developmental context. Incorporation of the OA into a persistent proatlantal trunk has not been described in acquired VA-branch collateral networks [7].

The classification of the present PPA is clarified by a recently published harmonised review [7] in this journal. Two partially conflicting systems coexist: the Lasjaunias course-based framework (type I: suboccipital sweep without C1 transverse foramen traversal; type II: ascent via the C1 transverse foramen to V3) and the Nakashima origin-based convention (type I: ICA origin; type II: ECA origin) [4, 13]. Within ECA-origin channels, Cohen et al. [14] distinguished a ‘spinal’ subtype (C1 transverse foramen traversal) from an ‘occipital’ subtype that bypasses transverse foramina and directly reaches the C1 vertebral groove, mimicking a type I suboccipital sweep. The PPA in the present case arises from the reconstituted ECA, forms a suboccipital loop giving rise to the terminal OA, and anastomoses with V3 and V2 extracranially without documented C1 transverse foramen traversal, consistent with type II occipital subtype (Cohen) and morphologically identical to the index case in Rusu et al. [7]: a 73 year-old woman with an ECA-origin type II occipital-subtype PPA giving rise to the OA and continuing as the extracranial VA before skull entry via the foramen magnum. The Vasovic et al. [1] framework would label the present vessel type I by course; however, Rusu et al. [7] demonstrate that this generates nomenclature overlap and recommend the Lasjaunias course-based system with Cohen’s subtypes as the most discriminating approach. We retain the designation type II ECA-origin, occipital subtype, throughout.

The present constellation carries additional relevance for thoracic outlet syndrome (TOS). When subclavian artery compression occurs in a patient harbouring a persisting CD connecting the subclavian artery to the distal ECA, compression reduces inflow into the CD, potentially compromising not only ECA-territory perfusion but also the vertebrobasilar circulation via the PPA [15]. Dynamic subclavian compression in TOS may precipitate vertebrobasilar insufficiency manifesting as dizziness, syncope, or visual disturbances with arm movement, rendering the clinical picture more complex than standard TOS [16]. This underscores the importance of pre-operative awareness of vascular anomalies to reduce iatrogenic risk during thoracic outlet and cervical vascular procedures.

## Conclusions

This report documents an adult right-sided constellation of (i) a persisting CD arising from the subclavian artery, (ii) proximal ECA critical occlusion/stenosis with distal ECA reconstitution, and (iii) a type II PPA forming two separate anastomoses to the VA (V3 and V2). To our knowledge, this is the first imaging evidence of a true ‘double-connection’ type II PPA to two VA segments. Thorough pre-procedural vascular imaging is advisable when cervical vascular variants are suspected, particularly before ECA ligation/embolisation or suboccipital surgical exposure.

## Data Availability

Data regarding this report can be obtained from the corresponding author on reasonable request.
